# Common Injuries Across Baseline, 6-Month, and 12-Month Assessments in CrossFit^®^ Athletes of Different Experience Levels

**DOI:** 10.3390/sports14050205

**Published:** 2026-05-18

**Authors:** Luiz Paulo Milares, Ricardo Luís Fernandes Guerra

**Affiliations:** 1 Sports Science Laboratory, Human Movements Sciences Department, Federal University of Sao Paulo, Santos 11010-908, São Paulo, Brazil; ricardo.guerra@unifesp.br; 2 Human Movement Sciences Department, Federal University of Sao Paulo, Santos 11015-020, São Paulo, Brazil

**Keywords:** athletic injuries, muscle strength, range of motion, asymmetry

## Abstract

Crossfit^®^ is a high-intensity interval training modality that combines weightlifting, aerobic exercises, and gymnastics. Although it has gained widespread popularity, it also presents a considerable injury rate without clarity on the extent to which experience categories exhibit distinct temporal patterns. This study identifies the most common injuries and their progression across CrossFit^®^ categories over 12 months. We defined injury as any Crossfit-related event requiring healthcare consultation and interrupting an athlete’s activity. An observational, longitudinal study was conducted with 102 participants categorized into three groups (n = 34): beginner, scale, and rx. An adapted injury index questionnaire was applied, and descriptive statistics were performed. Results showed that the most frequent injuries affected the shoulder and knee, with variations across the different athlete categories. Beginners exhibited the highest injury rates: knee (56%) and shoulder (35%). The scale group presented a greater concentration of shoulder injuries, whereas rx demonstrated the lowest injury incidence overall. Over the 12-month follow-up, 135 injuries were reported at baseline, decreasing to 116 at six months and 101 at the final evaluation. Dropout rates were 35% among beginners, 12% in the scale group, and 0% in the rx group. Crossfit-related injuries primarily affect the shoulders and knees, with a higher incidence in beginners. Future studies should investigate movement technique, strength, mobility, and limb dominance considering the overhead demands and the associated injury risk, in addition studies should examine training programming too

## 1. Introduction

Crossfit^®^, founded in the early 2000s by Glassman & Jenai [1], is a form of high-intensity interval training (HIIT) that incorporates elements of Olympic-style weightlifting, powerlifting, gymnastics, cardiovascular conditioning, and other functional movements [2,3], with exercises such as running, rowing, or cycling [4] in functional and constantly varied multi-joint movements [5], being adaptable to different levels of physical fitness [6]. Crossfit^®^ athletes are typically classified into three categories—beginner, scale, and rx—according to increasing physical demands in terms of intensity, duration, and complexity at each level [6,7,8,9,10]. Participation in Crossfit^®^ has been associated with improvements in strength VO_2_ max, endurance, body composition, aerobic capacity [11], and brain-derived neurotrophic factor (BNDF) levels [12]. Although Crossfit^®^ offers both physical and psychological benefits, it is frequently associated with a potential risk of musculoskeletal injuries [8,9].

Three main concerns have emerged regarding its safety: (a) execution of high-demand training sessions; (b) limited motor repertoire among individuals; and (c) a lack of qualified, certified, and experienced professionals [13]. These factors contribute to the increased risk of injuries during Crossfit^®^ practice as in all sports [14]. This combination of poorly structured or inadequately supervised exercise, excessive volume, and insufficient recovery can compromise movement quality, leading to premature fatigue, elevated perceived exertion, technical inefficiencies, and physical limitations such as reduced joint mobility, which may place CrossFit^®^ athletes at a higher risk of injuries [5,10,15,16]. The literature has explored CrossFit^®^ from various perspectives, including changes in physical fitness components, nutritional intake, supplementation with ergogenic aids, and injury occurrence [6,17,18]; however, evidence on how injuries behave across different athletes’ categories remains limited.

Thus, the hypothesis of this study is that, although injury risk is inherent to CrossFit^®^, injury patterns manifest differently across athlete categories. It remains unclear how injury patterns evolve across Crossfit^®^ categories over time. Despite the frequency of injuries reported in the literature, there is still a notable gap in understanding their impact on athletes’ progression or withdrawals. Monitoring injuries longitudinally, considering both experience levels and exposure time, may fill an important gap for athletes, coaches, and especially clinicians, contributing to safer practice by reducing the risk of recurrent injuries, surgical interventions, and long-term consequences. Elucidating these distinctions is essential for developing more precise and effective preventive strategies, tailored to the specific demands and vulnerabilities of each group.

This study identifies the most common injuries and their progression across CrossFit^®^ categories in three assessments: baseline, 6 months, and 12 months. Additionally, this study sought to analyze athlete progression between categories and to identify the factors associated with dropout from Crossfit^®^ practice.

## 2. Materials and Methods

### 2.1. Study Design

This longitudinal and descriptive study was conducted to identify the most common musculoskeletal injuries among Crossfit^®^ athletes across different training categories. This study was approved by the Human Research Ethics Committee (CEP 0084/2022) and followed the ethical standards of the Declaration of Helsinki. Data collection occurred over 12 months, with evaluations performed at three time points. Each evaluation session lasted approximately 10 min and took place between August 2022 and February 2025.

### 2.2. Participants and Eligibility Criteria

A total of 102 CrossFit^®^ athletes were recruited from multiple affiliated CrossFit^®^ training centers using a convenience sampling method. All participants provided written informed consent after being fully informed of the study purpose and procedures. Inclusion criteria were (1) age between 18 and 50 years, (2) active CrossFit^®^ participation in one of the three categories (beginner, scale, rx), and (3) have an injury. We recruited participants from ten CrossFit^®^ boxes in Brazil: eight located in São Paulo, SP, and two in Santos, SP. For rx classification, athletes were required to demonstrate the ability to perform all gymnastic and Olympic weightlifting movements regularly included in training sessions. Exclusion criteria included individuals over 50 years (master category) and those with any medical condition precluding participation.

Participants were categorized as follows:

Beginner: included individuals with limited experience in the modality who still required technical regressions, reduced loads, and simplified movement patterns (e.g., modified Olympic lifts, reduced plyometric demand, assistance in gymnastics).

Scale (“adapted”): consisted of participants who were able to perform most typical Crossfit^®^ movements but still trained with structured modifications, such as lighter loads, reduced volume, or substituted versions of higher skills (e.g., ring rows instead of pull-ups).

Rx (“as prescribed”): included participants who completed the workouts exactly as written, without load reduction or movement modification, meeting the full standards of execution (load, range of motion, and volume).

### 2.3. Injury Index Questionnaire

To identify musculoskeletal injuries, we used the questionnaire adapted and validated by Feito et al. (2018) [19] applied online through Google Forms. The questionnaire consists of biological variables, affected anatomical region, athlete category at the time of injury, and frequency of participation in training (weekly and number of workouts). We used the definition of injury according to Weisenthat et al. [20]: “any injury suffered while doing CrossFit^®^ that resulted in a consultation with a physician or health professional and that caused the athlete to stop or reduce their usual activity”.

### 2.4. Statistical Analysis

The required sample size for this study was calculated using G*Power software (version 3.1.9.2, Franz Faul, Universität Kiel, Germany), adopting an α error of 0.05 and a power of 0.8, in accordance with the previous study by Jusdado-García and Cuesta-Barriuso (effect size = 0.25, mean) [4]. Descriptive statistics were calculated for the three assessment time points (baseline, 6 months, 12 months) and for the three experience-based categories (beginners, scale, and rx). Continuous variables are presented as means and standard deviations, and—when clinically informative—as medians and interquartile ranges. Categorical variables are reported as absolute and relative frequencies. Because this study is observational and intended to characterize category- and time-specific patterns, descriptive analyses were prioritized to preserve clinical interpretability, considering unequal exposure and differential progression between categories over the 12-month follow-up.

In addition to a descriptive profile, we conducted an exploratory inferential analysis to examine whether injury incidence differed across categories and time. A Poisson regression model, using the number of injuries as the outcome and adjusting for training exposure (number of training sessions) was utilized. Incidence rate ratios (IRRs) with 95% confidence intervals were computed, and the results were summarized in a forest plot. All analyses were conducted using R Statistical Software (version 4.4.1) (R Core Team, 2024, Viena, Austria) [21]

## 3. Results

This study included both male and female athletes in comparable proportions; participants were classified into beginner, scale, and rx categories according to their self-reported training experiences and the complexity of exercises typically performed during workouts in accordance with their coach. Athletes were assessed at three time points (baseline, 6 months, and 12 months). A summary of demographic characteristics and group distribution is provided in Table 1.

### 3.1. Longitudinally, Changes in Athlete Classification Were Observed over the 12-Month Follow-Up

**Figure 1:**
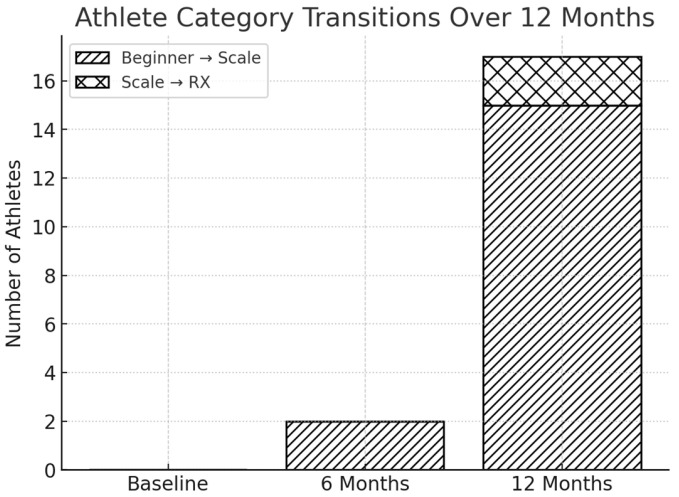
Progression of Crossfit^®^ athletes across competitive categories over 12 months.

No category shifts were observed at baseline, as this marked the initial assessment point. Over time, however, a progressive trend of advancement emerged, with athletes gradually moving toward higher levels of training complexity, as illustrated in Figure 1.

### 3.2. Regarding Dropout Rates During the 12-Month Follow-Up

Dropouts were observed exclusively in the beginner and scale groups, mostly associated with injury, pain, or time constraints. In contrast, no attrition occurred among rx athletes across the follow-up period, as shown in Table 2.

### 3.3. Training Characteristics and Injury Incidence Across Time in Crossfit^®^ Categories

Crossfit^®^ experience and training frequency displayed clear differences across categories, with more advanced athletes reporting longer practice history and slightly higher weekly volume. Over time, beginners showed a progressive increase in training frequency, approaching the levels of scale and rx participants, as summarized in Table 3.

### 3.4. Distribution of Number of Injuries and Sites over Time Across Crossfit^®^ Categories

**Figure 2:**
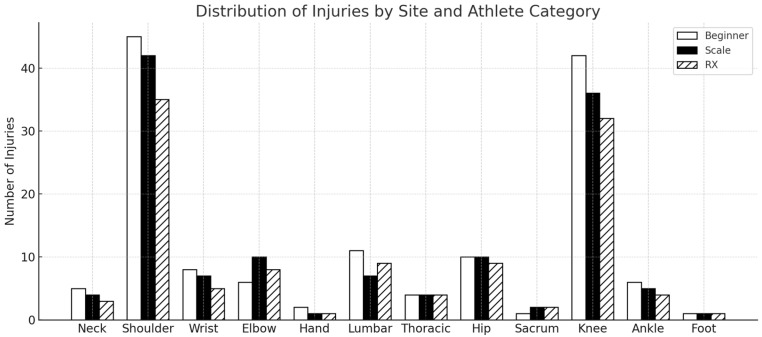
Distribution of injuries over one year in all athletes.

Across the follow-up, the total number of injuries progressively decreased, with a growing proportion of athletes reporting the number of injuries (Table 4). The anatomical distribution of injuries and their breakdown by athlete categories are shown in Figure 2. Complementarily, Table 5 details the distribution of injuries across body regions with each training group at baseline, 6 months, and 12 months, enabling comparison of region-specific involvement across time and groups.

### 3.5. Distribution of Injuries Across Three Evaluation Time Points in Beginner Athletes in Crossfit^®^

**Figure 3:**
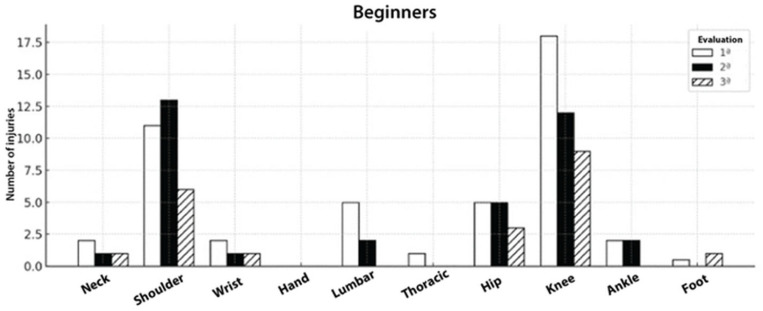
Injury counts by region at baseline (white), 6 months (black), and 12 months (hatched). Knee and shoulder injuries predominated with a downward trend over time; hip/lumbar were intermediate; other sides were infrequent.

Among beginners, knee and shoulder injuries were the most frequent across all time points. Although other regions were also affected, their occurrence was comparatively less relevant. Over the 12-month period, the overall number of injuries in this group showed a clear decline, as illustrated in Figure 3.

### 3.6. Distribution of Injuries Across Three Evaluation Time Points in Scale Athletes in Crossfit^®^

**Figure 4:**
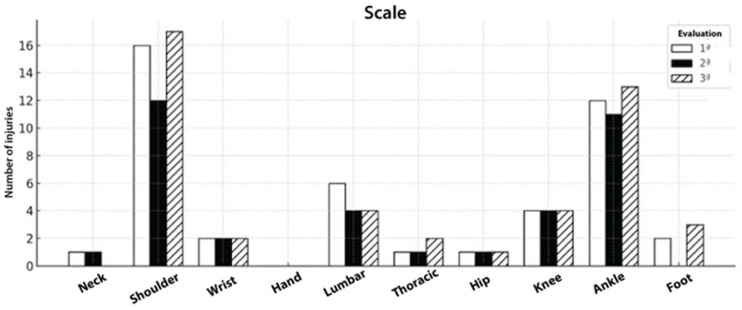
Injury counts by region at baseline (white), 6 months (black), and 12 months (hatched). Shoulder and knee injuries concentrated the burden, with a mild increase by 12 months; hip remained stable, lumbar decreased, and other sites were infrequent.

Among scale athletes, shoulder and knee injuries consistently represented the most affected regions across all time points; other sites such as the lumbar spine, hip and ankle were also reported, though with lower frequency, as illustrated in Figure 4.

### 3.7. Distribution of Injuries Across Three Evaluation Time Points in Rx Athletes in Crossfit^®^

**Figure 5:**
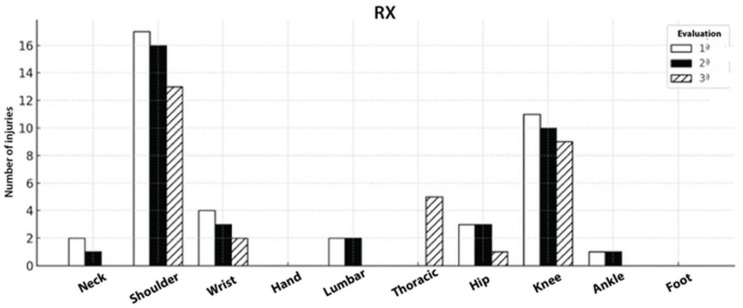
Injury counts by region at baseline (white), 6 months (black), and 12 months (hatched). Shoulder and knee predominated but declined over time, wrist/neck decreased; a small thoracic cluster appeared at 12 months; other sites were rare.

In the rx group, shoulder and knee injuries were the predominant sites across all assessments, with other regions such as the lumbar spine, hip, and wrist appearing less frequently. Over the 12-month follow-up, this group showed a gradual reduction in the total number of injuries, as illustrated in Figure 5.

### 3.8. Injury Incidence Rate Ratios by Time and Experience Level

**Figure 6:**
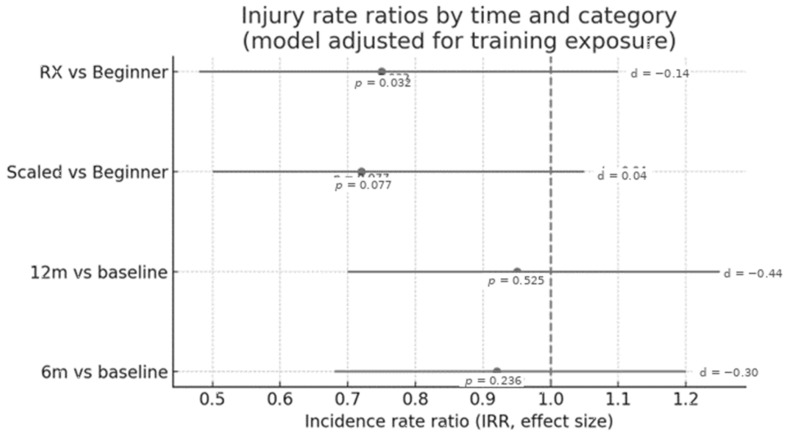
Incidence rate ratios (IRRs) and 95% confidence intervals for injury occurrence across time points and athlete experience levels. Estimates are derived from a Poisson regression model adjusted for training exposure (number of training sessions) and clustered by participants. The dashed vertical line represents the null value (IRR = 1.0). Only the comparison between rx and beginner athletes showed a statistically significant difference (*p* = 0.032), corresponding to lower injury incidence in the Rx group. Scaled vs. beginner and the time comparisons (6 months and 12 months vs. baseline) were not statistically significant.

The Figure 6 presents a forest plot of injury incidence rate ratios (IRR) estimated by a model adjusted for training exposure. The time point on each line represents the IRR estimate for the indicated comparison (category or time), and the horizontal bar corresponds to the confidence interval (CI) (as is standard for this type of graph). The dashed vertical line at IRR = 1.0 indicates no difference between groups/times; thus estimates to the left of 1.0 suggest lower rates of change in the compared condition, while estimates to the right suggest higher rates.

In comparisons by category, a significant difference was observed between Rx vs Beginner (*p* = 0.032), with an estimate below 1.0 indicating a lower rate of lesions in Rx compared to beginners, although with a small effect size (d = −0.14). Scale vs Beginner did not reach statistical significance (*p* = 0.077), with a trivial effect (d = 0.04). In comparisons by time relative to baseline, 6 months vs baseline (*p* = 0.236; d = −0.30) and 12 months vs baseline (*p* = 0.525; d = −0.44) did not show significant differences, with estimates close to 1.0 and intervals crossing the null line, suggesting stability of the lesion rate throughout the follow-up. Effect sizes (d) are reported on the right for each contrast agent.

## 4. Discussion

This is the first study to report musculoskeletal injuries across all three Crossfit^®^ experience levels over three assessment moments, revealing distinct patterns in injury incidence, anatomical distribution, and progression. It is notable that the data indicate a higher injury rate among beginner athletes. The adjusted model reinforces this observation by demonstrating that rx athletes had a significantly lower injury incidence (IRR ≈ 0.64; 95% CI 0.42–0.96; *p* = 0.032) compared to beginners, suggesting that greater technical proficiency and better self-regulation of training load among more experienced athletes may mitigate injury risk [9,17]. The training exposure and experience level, in either the 6-month or the 12-month assessment, differed significantly from baseline regarding injury incidence (IRR ≈ 0.93; 95 CI including 1.0; *p* > 0.23).

Shoulder and knee were the most frequently injured regions across all categories and time points. This pattern is consistent with previous evidence identifying these joints as particularly vulnerable during high-repetition functional training [15,22,23], especially in the case of the shoulder. The mastery of technical skills appears to be a fundamental factor modulating injury risk in these regions, as inadequate motor control or insufficient proficiency may increase joint loading during complex tasks. However, to understand the occurrence of these injuries in Crossfit^®^, it is essential to consider multiple interacting factors. Among them, practitioners’ experience and their progressive adaptation to the modality’s unique biomechanical demands play a central role, given that the movements performed in CrossFit^®^ differ substantially from those seen in traditional exercise programs [24].

While the shoulder and knee were the most frequently affected joints across all categories, other anatomical regions such as lumbar spine, wrist, and hip also showed relevant injury prevalence at lower frequencies. These findings are consistent with the previous literature reporting that multi-joint, compound movements under fatigue in Crossfit^®^ workouts place cumulative stress not only on primary movers but also on stabilizing structures and accessory joints [5,25]. The lumbar spine, in particular, was a recurrent site of injury among beginner and scale athletes, which may be associated with poor lumbopelvic control and insufficient core stability. Additionally, the biomechanical demands of deep squats commonly performed in Crossfit^®^—movements requiring deep hip flexion—often beyond parallel can heighten iliopsoas activation; when hip mobility is limited or technique is suboptimal, compensatory lumbar motion may increase compressive and shear loads on the lumbar spine, plausibly contributing to low-back injuries and even hip injuries.

Hip injuries, although less frequent, were reported across all groups and may be related to high-repetition squatting, snatch variations, and explosive hip extension patterns required in Olympic lifting. These exercises demand significant hip mobility and motor control, particularly in deep ranges of motion, which may not be fully developed in less experienced athletes. As we can see, the wrist was also a vulnerable region, especially in scale and rx groups, likely due to the demands of front rack positions, handstands, and cleans, which require not only wrist extension and load tolerance but also joint stability under dynamic conditions. Additionally, cumulative stress from repetitive and overhead transitions may exacerbate joint overload, especially when mobility restrictions are compensated by faulty movement patterns.

The findings of this study indicated that beginner athletes are those with the least experience, training frequency, and volume, presenting a profile that is distinct from that of other categories. It is essential that beginner athletes prioritize motor skill development, improve technical competence, and build the structural strength required to execute more complex movement patterns, as reflected in the higher injury and dropout rates observed among beginners (35%)—consistent with motor-learning demands and ongoing technical acquisition, this scenario contrast with that observed in the scale and rx categories, which recorded 12% and 0% dropout rates, respectively, suggesting that the experience acquired over time can act as a protective factor against injuries and contribute to permanence in the sport. According to Alekseyev et al. [3], adequate training of these skills can reduce the risk of injuries in the first years of practice.

In the context of Crossfit^®^, load progression and motor control are important as a preventive factor among beginner athletes. The knee was the most affected joint, possibly due to the difficulty in executing new movements. The practice of squat variations, such as clusters and thrusters, often involves external load and can result in osteomyoarticular overload if the technique is not adequately developed, which is why motor improvement is important in the initial phase of these athletes [9]. Bernsorff et al. [26] show in their study that the squat is the exercise most commonly associated with pain in Crossfit^®^, mainly for the back squat, while Gullet et al. [27] showed that the front squat presents better results in terms of muscle recruitment and lower compressive forces on the knee than the back squat.

CrossFit^®^ requires not only muscle strength but also good joint mobility for the efficient and safe execution of movements. Studies indicate that range of motion plays a fundamental role in performance and injury prevention [28,29], since movements such as deep squats, Olympic weightlifting, and gymnastics require a combination of strength and joint flexibility [30]. In addition, adequate mobility allows better movement mechanics, reducing compensations that can lead to ostemyoarticular overload [19]; therefore, Crossfit^®^ training programs should include specific exercises to improve mobility, ensuring not only strength gains but also biomechanical efficiency and a lower risk of injuries. This approach is essential to promote safer athletic progression, especially in less experienced athletes transitioning between training categories.

Several authors also emphasize the need to prioritize the development of motor skills, so that only then can the load be increased, and gradually [27,28,29], load and intensity control are essential factors for injury prevention. Studies show that excessive prioritization of training and volume without proper progression and individual adaptation can compromise movement quality and increase the risk of ostemyoarticular injuries [30,31,32]. Furthermore, when the load exceeds the practitioner’s physical capacity, there is a greater predisposition to inadequate movement patterns, which can generate joint overload and injuries, especially in more complex exercises such as Olympic weightlifting and gymnastics [13]. Thus, adequate training periodization respecting the principles of load progression and technique becomes essential for the safety and performance of practitioners.

Regarding upper limb injuries, there is a consensus in the literature that the shoulder is the most affected joint, as evidenced by several authors [8,9,15]. Including our research, the shoulder was prevalent in the three categories, during the three evaluation moments. This may occur due to the structural and functional complexity of the shoulder, which requires great mobility and stability to perform overhead movements, such as snatches, jerks, handstand push-ups, and kipping pull-ups, imposing significant demands on the glenohumeral joint, the rotator cuff complex, and the scapula [25], and cumulative stress from repeated overhead loading—particularly when stability or mobility is insufficient, may further increase risk. Lenz et al. [8] show that injuries occur notably with exercises involving barbells and bars. All of the thesis’s movements were performed in all Crossfit^®^ categories, but the variations (kipping and butterfly) were mostly performed by scale and rx, which could be one of the problems.

Pull-up is a multiplanar movement that utilizes muscle co-contraction to promote shoulder stability and is often used as an upper body strength and conditioning exercise. To perform the pull-up, the athlete needs a skill set that involves having an extremely strong upper limb to carry and move through a large range of motion overhead. Once again, the need for technical mastery combined with the execution of movements with a wide range of joint range is highlighted [33]. Common injuries among athletes performing these overhead tasks include impingement, tendonitis, and rotator cuff tears, which may be due in large part to the large forces required to trigger the pull-up movement.

The occurrence of injuries is an inherent factor in sports practice and is often associated with the physical and mechanical stress imposed on the body during the activity. Weisenthal et al. [20] found that approximately 19% of Crossfit^®^ practitioners reported at least one injury during their practice. These findings highlight the importance of implementing preventive strategies by professionals in the field, aiming not only to reduce injury rates but also improve athlete performance. In this context, technique plays a crucial role, especially among beginners, who have lower motor skills, and consequently, greater vulnerability to injuries. However, athletes in the scaled category should also receive attention, since they still have a considerable risk of injury. In contrast, more experienced athletes tend to have lower injury rates, possibly due to greater technical mastery and strength development, which contribute to the more efficient and safe execution of movements.

In the context of the aforementioned points, it is imperative to acknowledge the significance of technical aspects and factors such as muscle strength and joint mobility. These elements exert a substantial influence on the biomechanics of movements and the individual’s propensity to sustain injuries [3,29,30,31]. It is therefore imperative that these variables are analyzed in order to facilitate a more profound comprehension of injuries within the various categories of Crossfit^®^. This analysis should be given due consideration in future studies, as it will assist in the development of effective preventive strategies; future work should profile athlete and category-specific muscle strength and joint mobility and test these factors as mediating variables of injury risk.

This study presents some limitations that should be considered when interpreting the results. First, although the longitudinal design enables monitoring of injury occurrence over 12 months, the use of self-reported data via questionnaire may be subject to recall bias and variations in the perception of what constitutes an injury; second, the sample was drawn from different Crossfit^®^ boxes, which, despite reflecting real-world variability, introduced important heterogeneity in training protocols, coaching strategies, and program periodization. As a result, training volume, intensity, load progression, and supervision were not standardized or controlled, potentially influencing injury rates independently of athlete category. Additionally, the unequal distribution of participants across categories (beginner, scale, and rx) may have affected the statistical power to detect differences between groups. Despite these limitations, this study provides important insights into injury patterns among Crossfit^®^ athletes of varying experience levels and underscores the need for preventive approaches tailored to technical proficiency, physical capacity, and training context. These findings can inform the development of category-specific preventive programs and evidence-based recommendations for coaches and clinicians.

## 5. Conclusions

This study identified category-specific injury patterns in Crossfit^®^ athletes and demonstrated the importance of longitudinal monitoring for injury prevention. The injuries occur predominantly in the shoulder and knee joints, followed by the lumbar spine and hip. However, unlike previous reports that addressed injuries in a general way, our findings highlight that injury incidence and progression differ according to the athletes’ categories. Beginners exhibited the highest injury rates and dropout rates, mainly due to pain or injury, reflecting their lower technical mastery and limited neuromuscular adaptations. In contrast, scale athletes concentrated injuries in the shoulder, while RX athletes not only reported the lowest incidence but also showed no dropouts over 12 months, reinforcing the protective effect of technical proficiency and training experience.

Longitudinal monitoring revealed that, over time, athletes naturally progress between categories, and this evolution is accompanied by adaptations that reduce injury risk and favor continuity in practice. Thus, the distinction between categories is crucial to understanding the dynamics of injury in Crossfit^®^, since the behavior of athletes evolves as training exposure, motor learning, and technical efficiency consolidate. Future research should integrate objective biomechanical measures and real-time monitoring tools to improve preventive strategies and validate self-reported data.

## 6. Clinical Relevance

Injury prevention strategies in Crossfit^®^ should be adapted to each training category, emphasizing technique acquisition and load management for beginners, shoulder health and cumulative stress management for scaled athletes, and periodization for advanced practitioners. Beginners require greater emphasis on technical instructions, development of joint mobility, and progressive load control to reduce the risk of early overload. Scale athletes demand closer attention to the cumulative stress imposed on the shoulder complex, which, due to its spheroidal anatomy and high mobility, is inherently more susceptible to instability and musculoskeletal injuries. Rx athletes, in turn, benefit from appropriate periodization strategies that ensure long-term adaptations and protect against the consequences of chronic overload.

Safe progression also depends on coaches and health professionals designing training programs that consider athletes’ individual characteristics, applied loads, and the cumulative volume of workouts. Proper planning, aligned with continuous monitoring of technical execution and recovery, may represent a key factor in reducing injury incidence and promoting sustained performance across categories.

## Figures and Tables

**Table 1:** Characterization of Crossfit^®^ participants by category.

Variable	Beginner	Scale	Rx
Sex—Female (n/%)	24 (71%)	21 (62%)	10 (29%)
Sex—Male (n/%)	10 (29%)	13 (38%)	24 (71%)
Age—Mean ± SD	34.59 ± 8.48	33.53 ± 7.32	35.00 ± 7.48
Age—Median/IQR	33.00/14.75	34.00/9.50	36.00/10.50
Weight—Mean ± SD	74.70 ± 13.06	73.07 ± 12.68	76.80 ± 13.29
Weight—Median/IQR	73.00/23.25	73.00/16.50	75.00/16.00

**Table 2:** Dropout rates among Crossfit^®^ athletes by category and time point.

Category	Period	Number of Dropouts	Reason
Beginner	Baseline → 6 months	4	Injury/Pain
Beginner	6 → 12 months	7	Injury/Pain
Scale	Baseline → 6 months	2	Surgery (Shoulder SLAP), lack of time
Scale	6 → 12 months	2	Surgery (Lumbar arthrodesis), lack of time

**Table 3:** Descriptive measures of the variables of experience in Crossfit^®^, weekly frequency, number of workouts, and the number of injuries in the three groups at baseline, 6 months, and 12 months of assessment.

Variable		Beginner					Scale					RX				
		n	Mean	SD	Median	IIQ	n	Mean	SD	Median	IIQ	n	Mean	SD	Median	IIQ
Experience in CROSS		34	14.65	12.45	11.00	11.50	34	27.00	16.17	24.00	16.50	34	45.85	24.74	42.00	28.50
F (X/weekly)		34	4.27	1.26	4.00	2.00	34	4.82	0.83	5.00	0.75	34	5.24	0.89	5.00	1.00
Nº of workouts		34	4.94	2.35	5.00	2.00	34	6.12	2.35	5.00	1.00	34	6.32	2.66	5.00	1.75
Nº of injuries		34	1.38	0.70	1.00	0.75	34	1.41	0.66	1.00	1.00	34	1.29	0.58	1.00	0.00
Experience in Crossfit	**6 months**	30	22.03	12.57	19.00	13.00	32	31.97	15.56	30.00	14.50	34	51.88	24.70	48.00	28.50
F (X/weekly)		30	4.13	1.22	4.00	2.00	32	4.59	0.95	5.00	1.00	34	5.09	0.93	5.00	1.00
Nº of workout		30	4.90	2.56	4.00	2.00	32	6.06	2.34	5.00	1.50	34	5.97	2.34	5.00	1.00
Nº of injuries		30	1.17	0.87	1.00	0.75	32	1.19	0.64	1.00	0.25	34	1.21	0.88	1.00	1.00
Experience in Crossfit	**12 months**	22	28.50	14.11	23.50	14.00	30	38.30	15.93	36.00	16.50	34	57.71	24.49	54.00	28.50
F (X/weekly)		22	4.46	1.26	5.00	1.75	30	4.57	0.97	5.00	1.00	34	4.97	1.06	5.00	2.00
Nº of workout		22	5.41	2.77	5.00	1.00	30	6.07	2.46	5.00	2.50	34	5.91	2.39	5.00	1.75
Nº of injuries		22	0.91	0.61	1.00	0.00	30	1.07	0.64	1.00	0.00	34	0.91	0.90	1.00	2.00

SD: standard deviation; IIQ: interquartile range.

**Table 4:** Distribution of the number of injuries at the baseline, 6 months, and 12 months by groups.

Nº of Injuries	Basal			6 Months			12 Months		
	Beginner n = 34	Scale n = 34	RX n = 34	Beginner n = 30	Scale n = 32	RX n = 34	Beginner n = 22	Scale n = 30	RX n = 34
0				6 (20%)	3 (9%)	7 (21%)	5 (22%)	5 (17%)	14 (41%)
1	25 (74%)	23 (68%)	26 (76%)	16 (53%)	21 (66%)	16 (47%)	14 (64%)	18 (60%)	10 (29%)
2	5 (15%)	8 (24%)	6 (18%)	5 (17%)	7 (22%)	8 (24%)	3 (14%)	7 (23%)	9 (27%)
3	4 (11%)	3 (8%)	2 (6%)	3 (10%)	1 (3%)	3 (8%)	0 (0%)	0 (0%)	1 (3%)

**Table 5:** Distribution of injuries in different body regions between the training groups at baseline, 6 months, and 12 months.

Injuries	Baseline			6 Months			12 Months		
	Beginner n = 34	Scale n = 34	RX n = 34	Beginner n = 30	Scale n = 32	RX n = 34	Beginner n = 22	Scale n = 30	RX n = 34
Ankle	2 (5.9%)	2 (5.9%)	1 (2.9%)	2 (6.7%)	0 (0%)	1 (2.9%)	0 (0%)	0 (0%)	1 (2.9%)
Thoracic	1 (2.9%)	1 (2.9%)	0 (0%)	1 (3.3%)	1 (3.1%)	0 (0%)	1 (4.5%)	1 (3.3%)	0 (0%)
Sacrum	0 (0%)	1 (2.9%)	0 (0%)	0 (0%)	1 (3.1%)	0 (0%)	0 (0%)	1 (3.3%)	0 (0%)
Hip	3 (8.8%)	4 (12%)	3 (8.8%)	4 (13%)	4 (13%)	3 (8.8%)	2 (9.1%)	4 (13%)	1 (2.9%)
Wrist	2 (5.9%)	2 (5.9%)	4 (12%)	2 (6.7%)	2 (6.3%)	3 (8.8%)	1 (4.5%)	0 (0%)	2 (5.9%)
Shoulder	12 (35%)	17 (50%)	17 (50%)	13 (43%)	10 (31%)	16 (47%)	8 (36%)	9 (30%)	11 (32%)
Hand	1 (2.9%)	0 (0%)	0 (0%)	1 (3.3%)	0 (0%)	0 (0%)	1 (4.5%)	0 (0%)	0 (0%)
Lumbar	3 (8.8%)	6 (18%)	2 (5.9%)	1 (3.3%)	4 (13%)	3 (8.8%)	0 (0%)	4 (13%)	3 (8.8%)
Knee	19 (56%)	12 (35%)	11 (32%)	11 (37%)	11 (34%)	10 (29%)	7 (32%)	9 (30%)	8 (24%)
Elbow	0 (0%)	3 (8.8%)	4 (12%)	0 (0%)	4 (13%)	4 (12%)	0 (0%)	3 (10%)	4 (12%)
Cervical	2 (5.9%)	0 (0%)	2 (5.9%)	0 (0%)	1 (3.1%)	1 (2.9%)	0 (0%)	1 (3.3%)	1 (2.9%)
Foot	2 (5.9%)	0 (0%)	0 (0%)	0 (0%)	0 (0%)	0 (0%)	0 (0%)	0 (0%)	0 (0%)

## Data Availability

The data presented in this study are available on request from the corresponding author due to ethical and privacy restrictions.

## Abbreviations

The following abbreviations are used in this manuscript:

BNDF	Brain-derived neurotrophic factor
CEP	Brazilian Research Ethics Committee
HIIT	high-intensity interval training
IQR	Interquartile range
SD	Standard deviation
SLAP	Superior labrum from anterior to posterior
VO_2max_	maximal oxygen uptake
